# Hypoxia and the regulation of myeloid cell metabolic imprinting: consequences for the inflammatory response

**DOI:** 10.15252/embr.201847388

**Published:** 2019-03-15

**Authors:** Pranvera Sadiku, Sarah R Walmsley

**Affiliations:** ^1^ Centre for Inflammation Research The Queen's Medical Research Institute University of Edinburgh Edinburgh UK

**Keywords:** hypoxia‐inducible factors, hypoxia, immunometabolism, macrophages, neutrophils, Immunology, Metabolism

## Abstract

Inflamed and infected tissue sites are characterised by oxygen and nutrient deprivation. The cellular adaptations to insufficient oxygenation, hypoxia, are mainly regulated by a family of transcription factors known as hypoxia‐inducible factors (HIFs). The protein members of the HIF signalling pathway are critical regulators of both the innate and adaptive immune responses, and there is an increasing body of evidence to suggest that the elicited changes occur through cellular metabolic reprogramming. Here, we review the literature on innate immunometabolism to date and discuss the role of hypoxia in innate cell metabolic reprogramming, and how this determines immune responses.

**Glossary**
2‐DG: 2‐deoxyglucose
Ap3A: diadenosine triphosphate
ATP: adenosine triphosphate
CARKL: carbohydrate kinase‐like protein
Cx43: connexin 43
DCs: dendritic cells
FADH_2_: flavin adenine dinucleotide
FIH: factor Inhibiting HIF
G6P: glucose‐6‐phosphate
G6PT: glucose‐6‐phosphate (G6P) transporter
GAPDH: glyceraldehyde 3‐phosphate dehydrogenase
GSD‐Ib: glycogen storage disease type Ib
HID: hyper‐IgD syndrome
HIF: hypoxia‐inducible factor
LPS: lipopolysaccharide
NADH: nicotinamide adenine dinucleotide
NADPH: nicotinamide adenine dinucleotide phosphate
NETs: neutrophil extracellular traps
NO: nitric oxide
NOX2: NADPH oxidase 2
OPA1: optic atrophy 1
PDH: pyruvate dehydrogenase
PDK1: pyruvate dehydrogenase kinase 1
PHDs: prolyl hydroxylase domain enzymes
PKM2: pyruvate kinase M2
PNS: peripheral nervous system
PPP: pentose phosphate pathway
ROS: reactive oxygen species
TAMs: tumour‐associated macrophages
TCA: tricarboxylic acid cycle
VHL: von Hippel Lindau

## Introduction

A key feature of immune cells is their ability to infiltrate and function in hypoxic tissues, enabling appropriate responses to damage and infection. Hypoxia primes innate cells for the inflammatory response and prolongs survival in numerous types of innate immune cells including neutrophils, monocytes and eosinophils 1, 2, 3, 4. HIF‐mediated innate immune responses are discussed in detail in a review by Harris *et al* 5. Both hypoxia and the activation of innate cells by inflammatory stimuli result in the induction of the master regulator of oxygen homeostasis, hypoxia‐inducible factor‐1 (HIF‐1) 6. HIF‐1 induction mediates a response through the regulation of expression of hundreds of downstream genes involved in diverse biological pathways, ranging from erythropoiesis to metabolism 7, 8, 9. In mammals, HIF‐1 exists as a heterodimeric complex composed of two subunits, HIF‐1α (oxygen‐sensitive) and HIF‐1β (constitutive) 10, 11. Hypoxia‐inducible factor activity is regulated by a family of oxygen sensing proteins known as prolyl hydroxylase domain enzymes (PHD1‐3) and asparaginyl hydroxylase enzyme factor inhibiting HIF (FIH) 11, 12, 13, 14. Prolyl hydroxylase domain enzymes post‐translationally modify the HIF‐1α subunit through hydroxylation of two conserved proline residues, in an oxygen‐dependent manner. Hydroxylation destabilises HIF‐1α by mediating binding to von Hippel Lindau (VHL) protein E3 ligase complex followed by ubiquitination and proteasomal degradation 15, 16. In order to function, in addition to oxygen, PHD and FIH proteins also require Fe^2+^, ascorbic acid and the tricarboxylic acid (TCA) cycle intermediary α‐ketoglutarate 17. Furthermore, these proteins have been shown to be inhibited by the accumulation of downstream TCA intermediaries succinate and fumarate in studies using cancer cells and *in vitro* studies where cells were exposed to high concentrations of TCA cycle intermediates 18, 19. These findings highlighted a direct link between metabolite availability and the regulation of HIF‐1 activity. To date, there are a limited number of studies investigating metabolic regulation via TCA cycle metabolites under physiological oxygen conditions in myeloid cells *in vivo*. In hematopoietic stem cells, fumarate breakdown has been identified as a critical regulatory mechanism for stem cell maintenance and hematopoietic differentiation in normal hematopoiesis and leukaemia propagation 20. A second study by Tyrakis *et al* 21 demonstrated the accumulation of the metabolite 2‐hydroxyglutarate in mouse CD8^+^ T cells in response to T‐cell receptor triggering and showed that it occurs through a HIF‐1α‐dependent mechanism with subsequent epigenetic changes. In *in vitro* assays utilising activated macrophages, succinate accumulation has been shown to be important in the regulation of downstream cellular responses 22. It remains to be seen whether other TCA cycle metabolites act as immunoregulatory molecules in neutrophils and macrophages in normal physiology and during inflammatory hypoxia in the tissues.

In this review, we focus on the effects of hypoxia on neutrophil and macrophage metabolic regulation. For a more general overview of the HIF pathway relevance for metabolism in all immune cells, see the review by Krzywinska and Stockmann 23.

## Regulation of metabolism by hypoxia

For adequate function, innate immune cells require readily available ATP, redox buffering capacity and biosynthetic precursors. Under normoxic oxygen levels, most cells metabolise glucose into pyruvate via glycolysis. Pyruvate then enters the mitochondria and can be converted into acetyl‐coenzyme A, which is further oxidised in the TCA cycle. This process, known as aerobic respiration, generates the reducing equivalents nicotinamide adenine dinucleotide (NADH) and flavin adenine dinucleotide (FADH_2_), which donate electrons to the electron transport chain and fuel oxidative phosphorylation. Aerobic respiration generates high levels of energy stores in the form of adenosine triphosphate (ATP) molecules. It was initially thought that hypoxic cells relied solely on anaerobic glycolysis, a phenomenon termed the “Pasteur effect” 24. This phenomenon was used to describe the elevated conversion of glucose to lactate as a result of HIF‐activated glycolysis and was thought to be a result of a decline in aerobic respiration, a passive process resulting from oxygen deprivation. However, mounting evidence has revealed that hypoxia actively regulates metabolic pathways, and in addition to upregulating glycolytic flux, it also suppresses the TCA cycle and the mitochondrial respiratory chain 25. A study by Kim *et al* 26 has demonstrated that mitochondrial respiration in hypoxic cells causes leakage of electrons in the transport chain leading to increased reactive oxygen species (ROS) levels and cell damage. Pyruvate dehydrogenase (PDH) inhibition through HIF‐1‐mediated pyruvate dehydrogenase kinase 1 (PDK1) induction was shown to prevent cell damage by attenuating mitochondrial respiration and shunting metabolism to glycolysis. This subsequently results in the maintenance of ATP levels and restored homeostasis.

## The impact of hypoxia on immunometabolism

Innate immune cells eradicate pathogens and convey signals to the adaptive immune system in turn regulating its response. Innate cells may be resident cells or cells that migrate through a range of oxygen tensions before reaching very hypoxic environments such as inflamed or infected tissues. These cells therefore require oxygen‐independent mechanisms of energy generation. Hypoxia results in the activation of numerous innate immune cells, and the HIF pathway has been shown to play key roles in both effective and pathological immunity 27. Hypoxia‐inducible factor activation can occur under normoxia, and this allows the initiation of an inflammatory response before the tissue becomes hypoxic. Gram‐negative bacterial product lipopolysaccharide (LPS), ROS and reduced cellular iron have all been shown to regulate HIF‐1 expression under normoxic conditions through upregulation of *HIF‐1α* transcript expression, HIF‐1α stabilisation or inhibition of PHDs, respectively 28, 29, 30.

### Neutrophils

Neutrophils constitute around 60% of the circulating leucocytes. They are short‐lived polymorphonuclear cells and the first to migrate to injured or infected tissue sites where oxygen availability is limited. Their role is to combat bacterial infections through phagocytosis, respiratory burst activity, the release of granule contents and extracellular traps. Hypoxia prolongs neutrophil survival by inhibiting programmed cell death and is accompanied by a time‐dependent induction of key glycolytic enzymes glyceraldehyde 3‐phosphate dehydrogenase (GAPDH) and triosephosphate isomerase‐1 1. Murine studies on *HIF‐1α* knockouts have demonstrated that the HIF‐1α protein is essential for myeloid cell infiltration and activation. HIF‐1α inactivation results in a reduced cellular ATP pool, impairment of myeloid cell aggregation, motility, invasiveness and bacterial killing 31, 32. Although HIF‐2α is also upregulated in neutrophils in response to hypoxia, it is thought to play a key role in the resolution phase of inflammation and its effects on metabolism are not understood 33.

Neutrophils contain very few mitochondria and as such are thought to lack the capacity for mitochondrial respiration 34. In keeping with this, it has been shown that the inhibition of oxidative phosphorylation has little effect on the oxygen consumption rate of neutrophils 35. Neutrophils rely heavily on glycolysis even in the presence of oxygen 36, 37. In keeping with this, neutrophil activation results in increased glucose transport and intrinsic activation of glucose transporter molecules 38.

Glycolysis provides a very rapid supply of energy and is well suited to the function of recruited innate immune cells. As early as in the 1950s, Borregaard and Herlin demonstrated that glycolysis is used to fuel neutrophil phagocytosis in both the presence and absence of glucose 39. The ATP required for the phagocytosis of zymosan particles was almost exclusively generated through either glucose uptake or glycogenolysis in a glucose‐deplete setting. This finding was further supported by a study investigating the inhibition of glycolysis by 2‐deoxyglucose (2‐DG) on guinea pig neutrophils, where treatment of cells with the inhibitor led to an impairment of the phagocytosis of C3‐ and IgG‐bound particles 40. The effects of the metabolic substrates fatty acids and glutamine have been studied to a lesser extent, but both have also been shown to play roles in neutrophil development and homeostasis. While autophagy‐derived free fatty acids and their oxidation are required for appropriate neutrophil differentiation, the role of glutamine is critical in regulating inflammatory processes through delaying spontaneous apoptosis 41, 42.

A shunt into the pentose phosphate pathway (PPP), a side pathway of glycolysis, ensures that the neutrophil has a supply of nicotinamide adenine dinucleotide phosphate (NADPH) cofactor for respiratory burst via NADPH oxidase 2 (NOX2) activity 43. The importance of the pentose phosphate pathway in neutrophil reactive oxygen species production has been established in studies on patients with glucose‐6‐phosphate dehydrogenase deficiency, who have abnormal neutrophil function and increased susceptibility to infections 44. These patients have neutrophils with very low levels of NADPH, unable to produce normal quantities of superoxide species. *In vitro*, inhibition of the pentose phosphate pathway using the 6‐phosphogluconate‐dehydrogenase inhibitor 6‐aminonicotinamide abrogated bacterial peptide fMLF and phorbol myristate acetate induced NADPH production and superoxide production 45.

Neutrophil activation results in the production of extracellular traps or fibres consisting of granule proteins and chromatin 46. Neutrophil extracellular traps (NETs) degrade virulence factors and kill bacteria. It has been reported that NETosis, the form of cell death characterised by the release of NETs, is dependent on NADPH oxidase, as patients with chronic granulomatous disease who carry mutations in NADPH oxidase are unable to produce NETs 47. Conversely, NETs have also been implicated in the pathogenesis of autoimmune and inflammatory disorders 48. The formation of neutrophil extracellular traps is heavily dependent on glucose substrate and to a lesser extent on glutamine 49. Moreover, a study by Azevedo *et al* 50 observed that a shift to the pentose phosphate pathway and not mitochondrial ROS is necessary for the generation of NETs. Although mitochondrial ROS are not effective in producing NETs, a very recent study claims that the mitochondrial inner membrane protein optic atrophy 1 (OPA1) is important for the anti‐microbial defence of neutrophils in an *in vivo* model of *P. aeruginosa* lung infection 51. OPA1 was shown to function through the control of the mitochondrial electron transport complex I and in turn regulate NAD^+^ availability. NAD^+^ is required for the enzymatic activity of the glycolytic protein glyceraldehyde‐3‐phosphate dehydrogenase. Opa1‐deficient neutrophils had lower NAD^+^ levels resulting in a reduced glycolytic rate and a decline in ATP production.

In addition to increased uptake and affinity for glucose during neutrophil activation, neutrophils also contain and upregulate glucose stores in the form of the macromolecule glycogen 38, 52, 53, 54. Hypoxia is known to promote glycogen accumulation through HIF‐1‐mediated induction of glycogen synthase 1 55. We have recently demonstrated the consequences of HIF‐1α stabilisation for neutrophilic inflammation. Our work has shown that PHD2‐deficient neutrophils have enhanced survival, chemotaxis and functional reserve capacity resulting in an exaggerated inflammatory response that is detrimental to the host 56. This disordered neutrophilic response is driven by increases in both glycolytic flux and glycogen storage. Importantly, non‐PHD‐isoform‐selective inhibition using Molidustat also replicated the Phd‐2‐deficient phenotype augmenting neutrophilic inflammation via increased extracellular acidification rate (an indirect measure of glycolysis) and ATP levels 56. With numerous PHD protein inhibitors being used for the pharmacological treatment of anaemia associated with chronic disease and in late phase clinical trials 57, understanding the consequences of pan hydroxylase inhibition for immunometabolism and therefore innate cellular responses will be crucial for determining when and how these inhibitors are administered to limit adverse effects.

More broadly, the importance of glucose homeostasis for neutrophil function is supported by study of patients with glycogen storage disease type Ib (GSD‐Ib). In this setting, defective glucose‐6‐phosphate (G6P) transporter (G6PT) activity is associated with impaired neutrophil respiratory burst, chemotaxis and calcium mobilisation, resulting in an increased susceptibility to infection 58, 59, 60. Furthermore, mice lacking glucose‐6‐phosphatase‐b (G6Pase‐b) also demonstrate an increased susceptibility to bacterial infection with elevated neutrophil apoptosis responses as a consequence of dysregulated activation of the HIF‐1α/PPAR‐γ pathway 59, 60. Together therefore, these data highlight the importance of the tight regulation of intracellular glucose shunting for effective neutrophil responses.

**Figure 1:**
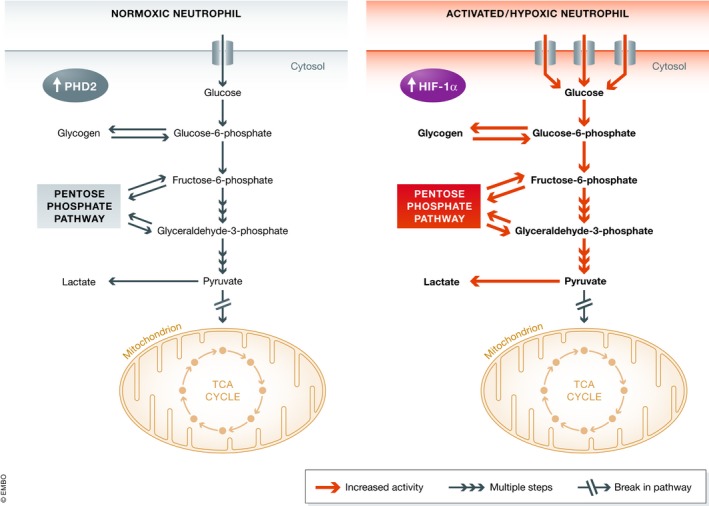
Neutrophil metabolic changes during exposure to hypoxia and activation Under normoxic conditions (left), neutrophils express high levels of PHD2 which result in the targeting of HIF‐1α for proteasomal degradation. In this setting, neutrophil homeostasis relies on the glycolytic pathway. Neutrophils further increase both their glycolytic and pentose phosphate pathway flux in response to hypoxic culture and stimulation (right). In addition, they also upregulate the influx of extracellular glucose and glycogen storage. The neutrophil TCA cycle is a dysfunctional cycle with only partial activity.

Besides the intracellular metabolic regulation, neutrophils have also been reported to release metabolites to the external environment. It has long been known that neutrophils release both AMP and ATP at inflammatory sites 61, 62. Adenosine triphosphate is released both through connexin 43 (Cx43) hemichannels and through vesicular nucleotide transporters 62, 63. ATP secretion results in autocrine stimulation of these cells through purinergic receptors. Additionally, it directs the cell orientation and is rapidly hydrolysed 64. There is some evidence from *in vitro* models of endothelial barrier function and neutrophil‐endothelial adhesion to indicate that neutrophils secrete ATP in response to hypoxia and inflammation 65. In this setting, it was demonstrated that neutrophil‐derived ATP signals through endothelial adenosine receptors to promote endothelial barrier function and attenuates PMN‐endothelial adhesion 65. In a second study by Eltzschig *et al* 66 *,* endogenous neutrophil adenosine produced during hypoxia was shown to reduce neutrophil accumulation implicating this pathway as a mechanism for attenuating excessive neutrophil inflammation. More recently, there is novel evidence for activated neutrophils acting as a source of diadenosine triphosphate (Ap3A) and ensuring an additional supply of adenosine during mucosal inflammation resolution 67. These studies highlight the metabolic crosstalk between neutrophils and other tissues and provide support for the role of neutrophils in regulating barrier formation and wound healing responses. A summary diagram of the metabolic changes occurring during exposure to hypoxia and activation of neutrophils is shown in Fig 1.

### Macrophages

Monocytes are short‐lived mononuclear phagocytes circulating in the bloodstream. They contribute to and replenish tissue‐resident phagocytes by giving rise to monocyte‐derived macrophages and dendritic cells (DCs). Macrophages play a central role in the innate immune response and are characterised by considerable functional diversity and plasticity 68. These cells are recruited to sites of injury or infection to either promote or reduce inflammation through the phagocytosis of bacteria and efferocytosis 69. In addition, they help to repair the tissue damage caused by the innate immune response. Macrophage accumulation occurs in numerous hypoxic tissues such as arthritic joints, atherosclerotic plaques and malignant tissues, implicating these cells in these diseases. Both human monocytes and macrophages markedly upregulate HIF‐1α levels during *in vitro* exposure to hypoxic environments as well as to LPS exposure 70, 71. Furthermore, human macrophages have also been shown to upregulate their HIF‐2α levels under hypoxia 71. HIF‐1α deficiency in macrophages displays defects in key cellular functions including motility, aggregation and invasion 31. By contrast, HIF‐2α‐deficient macrophages lose 50% of their migratory and invasive capacity 72.

Over 40 years ago, Hard made the observation that activated macrophages were more glycolytic and had a decreased oxygen consumption rate 73. Twenty years later, in a study by Newsholme *et al* 74 it was shown that most of the glucose in activated macrophages is utilised for conversion into lactic acid and a very low level was being used for oxidative phosphorylation. These findings were paralleled by increases in the expression of glycolytic enzyme hexokinase and the PPP enzyme glucose‐6‐phosphate dehydrogenase 75.

Activated macrophages are commonly divided into two polarised states or phenotypes, the classically activated M1 and the alternatively activated M2 76, 77. M1‐polarised macrophages are reported to have induced HIF‐1α expression, whereas M2 macrophages demonstrate upregulated HIF‐2α levels 78. In keeping with this finding, myeloid‐specific overexpression of HIF‐1α in mice resulted in a hyper‐inflammatory state characterised by the upregulation of M1 markers and was associated with increased glycolytic activity 70. As with neutrophils, the PHD2 isoform is the main regulator of the glycolytic reprogramming of macrophages demonstrated by gene knockout studies in mice 79. A role for PHDs in macrophage function was demonstrated in a hind‐limb ischaemia study, where PHD2 haplodeficiency in mice was sufficient to skew macrophages to an M2 phenotype without affecting HIF‐1α levels leading to a pro‐arteriogenic phenotype in turn preventing hind‐limb ischaemia in a murine model 80, 81.

Over the last few years, it has become increasingly apparent that the two divergent macrophage phenotypes are associated with differing metabolic states. M1 polarisation results from activation of interferon‐γ or toll‐like receptor activation by LPS. In this setting, both macrophages and dendritic cells increase the expression of the nitric oxide synthase generating large amounts of nitric oxide, which inhibits mitochondrial respiration and is utilised for bacterial killing 81, 82. In contrast, M2 polarisation is a consequence of exposure to IL‐4 or IL‐13 68. The M2 phenotype is characterised by fatty acid oxidation and mitochondrial biogenesis via STAT6 and PGC‐1β and reduced nitric oxide levels 83, 84. Inhibition of the mitochondrial respiratory chain in M2 macrophages not only blocks the M2 phenotype but also drives the cells to an M1 phenotype 83. In addition, M2 macrophages have upregulated levels of arginase 1 driven by HIF‐2α 78, 85. Arginase 1 is an enzyme, which competes with inducible nitric oxide for the common substrate L‐arginine.

Macrophage activation via LPS exposure induces dramatic metabolic reprogramming. As mentioned earlier, M1 macrophages undergo a switch from generating ATP by oxidative phosphorylation to glycolysis. The dramatic changes in the metabolic activity of M1 macrophages are also regulated by the attenuation of pyruvate kinase M2 (PKM2) levels. Activated PKM2 can bind to HIF‐1α and prevent it from inducing the IL‐1β promoter activity 22. The metabolic shift in M1 macrophages resembles the Warburg effect first reported in tumour cells and is associated with increased levels of the TCA cycle intermediary succinate due to high glutaminolysis activity 86, 87. The TCA cycle in these cells is broken at two points: after citrate and after succinate 88. The effects of succinate are exerted in three ways, firstly by inhibiting PHD proteins and thus leading to the stabilisation of HIF‐1α 89. Secondly, the elevated levels of succinate result in increased protein succinylation states, glycolytic targets of which include glyceraldehyde 3‐phosphate dehydrogenase (GAPDH) and malate dehydrogenase 87. It is not known whether this affects the enzymatic activity of these proteins. Thirdly, the oxidation of succinate in M1 via succinate dehydrogenase macrophages generates mitochondrial ROS and it has been reported that this is crucial for determining the inflammatory phenotype 90.

**Figure 2:**
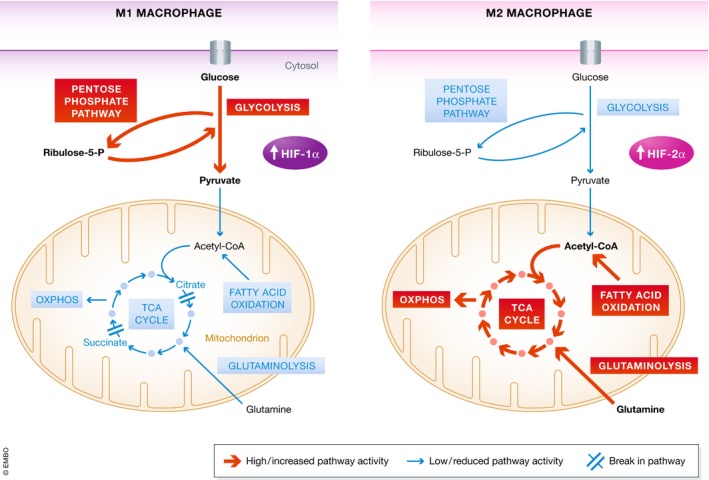
Macrophage metabolic profiles of M1‐ and M2‐polarised cells M1 activated macrophages are reported to have elevated HIF‐1α expression associated with an increase in the utilisation of the glycolytic and pentose phosphate pathway (left). Conversely, these cells demonstrate a reduced activity of mitochondrial respiration with the TCA cycle being broken at two points: after citrate and after succinate. Alternatively, activated M2 macrophages (right) show upregulated HIF‐2α expression and are characterised by increased fatty acid oxidation and mitochondrial respiratory chain activity.

There is evidence to suggest that macrophage polarisation can be influenced by changes in glucose metabolism and PPP activity 91, 92. The carbohydrate kinase‐like protein (CARKL) was identified as a non‐protein kinase that is rapidly downregulated upon LPS stimulation in mice and humans and directs carbon reshuffling between glycolysis and the PPP. It was shown to play an important role in sensitising macrophages to an M2 polarisation by shifting glucose utilisation from the PPP to glycolysis. In the M1 macrophages on the other hand, elevated PPP activity generates NADPH important both for ROS and for NO synthesis 93. Another M1‐regulated metabolic switch occurs through increased expression of PFKFB3, an isoform of 6‐phosphofructo‐2‐kinase/fructose‐2,6‐bisphosphatase (PFK‐2), which increases the levels of the metabolite fructose‐2,6‐bisphosphate 94, 95. This metabolite acts by activating the glycolytic enzyme 6‐phosphofructo‐1‐kinase. In addition, the attenuated levels of fatty acid oxidation and mitochondrial respiratory chain activity in both dendritic cells and M1 macrophages are achieved through the inhibition of AMPK 96, 97. A summary diagram showing the regulation of different metabolic pathways in M1 and M2 macrophages is shown in Fig 2.

Substrate availability can also regulate macrophage polarisation. Mechanistic target of rapamycin (mTOR) signalling has been linked to macrophage polarisation and is likely to play a role in regulating responses to substrate availability 98. For example, both mTORC1 and Lamtor1, a lysosomal adaptor protein which forms an amino acid sensing complex with lysosomal vacuolar‐type H^+^‐ATPase and is the scaffold for amino acid‐activated mTORC1, were shown to be required for M2 polarisation 99. Jha *et al* 88 also demonstrated that glutamine deprivation can negatively affect M2‐polarised macrophages but not M1. This was linked to the glutamine deprivation changes exerted on the TCA cycle. Moreover, the UDP‐GlcNac biosynthesis pathway was also highlighted as being important for M2 activation and was proposed by the authors to function by facilitating protein folding 88.

**Figure 3:**
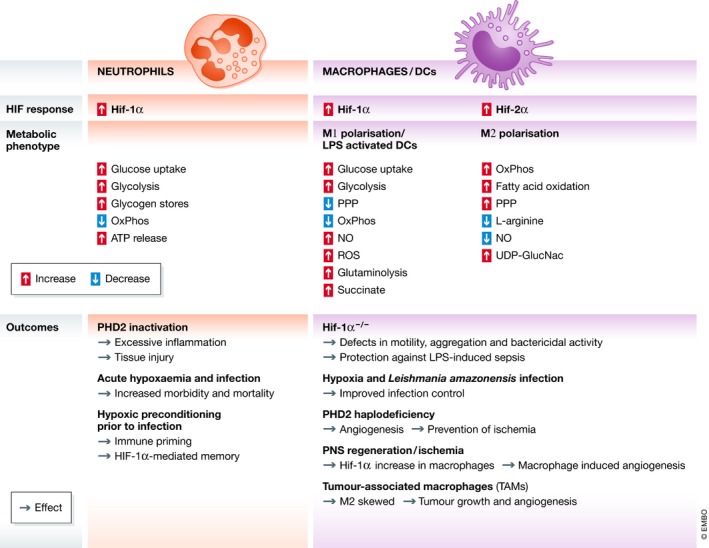
A table summarising the metabolic phenotypes and physiological outcomes documented to date linking hypoxia to myeloid cell metabolism The metabolic changes associated with elevated Hif‐1α and Hif‐2α expression are shown for neutrophils (left, orange) and macrophages/DCs (right, purple). The inactivation of both PHD2 and HIF‐1α protein is reported to result in dysregulated neutrophil and macrophage function in murine models.

Several studies have reported the influence of hypoxia and HIF expression on macrophage responses. For example, hypoxia has been shown to improve the control *Leishmania amazonensis* infection 100. Both HIF‐1α and HIF‐2α were shown to be upregulated in the macrophages in this model, and ROS was proposed as the mechanism of the parasite killing. In another study investigating the effect of macrophages on peripheral nervous system (PNS) regeneration, macrophages were shown to respond to hypoxia by migrating to the wound and inducing angiogenesis at the severed region 101. In tumour models, macrophages can constitute up to 50% of the tumour mass 102. They accumulate in poorly vascularised and hypoxic environments and are usually associated with a poor prognosis. Tumour‐associated macrophages (TAMs) are thought to function by promoting tumour cell proliferation and angiogenesis 103. Although TAMs are M2‐skewed, whether they have the same metabolic phenotype of an M2 macrophage remains unknown. A summary diagram of the metabolic changes during hypoxic exposure and activation of immune cells and the inflammatory outcomes is shown in Fig 3.

## Immune memory and hypoxia

Recently, there have been numerous reports describing transcriptional and epigenetic changes within immune cells and linking these to the inflammatory response. The phenomenon termed “innate immune memory” has been demonstrated in monocytes, macrophages, natural killer cells and neutrophils 104. In a study by Netea *et al*, the metabolite mevalonate was shown to mediate long‐term reprogramming in monocytes in response to β‐glucan via the activation of IGF1 receptor and mTOR subsequently resulting in histone modifications 105. Multiple major metabolic pathways including glucose metabolism, glutaminolysis and the cholesterol synthesis pathway are upregulated in β‐glucan‐trained macrophages 106, 107. HIF is central to β‐glucan training, and mice lacking myeloid‐specific HIF‐1α lack the ability to induce trained immune responses to sepsis 106. β‐glucan is a component of fungal cell walls and is associated with stable changes in histone trimethylation at H3K4, which confer protection from secondary lethal infection through priming of the production of pro‐inflammatory cytokines 108. A second metabolite known to act as an immunometabolite is 2‐hydroxyglutarate. This metabolite is known to accumulate in cancer cells with gain‐of‐function mutations of the isocitrate dehydrogenase 1/2 genes 109. *In vitro*, 2‐hydroxyglutarate levels increase in response to hypoxia and T‐cell receptor triggering through a hypoxia‐inducible factor 1‐alpha (HIF‐1α)‐dependent mechanism 21. The authors of this study proposed that 2‐hydroxyglutarate functions by inhibiting 2‐oxoglutarate‐dependent dioxygenases that demethylate histones or oxidise 5‐methylcytosine in DNA as 2‐hydroxyglutarate treatment resulted in higher methylation in CD8^+^ T cells. In a second study, Burr *et al* 110 showed that 2‐hydroxyglutarate inhibits both HIFα prolyl hydroxylases (PHDs) and TET 2‐oxoglutarate‐dependent dioxygenases. Conversely, hypoxic preconditioning has also been shown to result in innate immune memory in a HIF‐1α‐dependent manner. Exposure to prolonged hypoxia prior to infection protects against the increase in morbidity and mortality that is observed with acute hypoxia 111. These changes are mediated, in part, by neutrophils and associated with reduced HIF‐1 pathway and metabolic gene expression. It is not known whether the neutrophil‐driven memory effects are driven by immunometabolites. Further work is required in order to determine the role of immunometabolites in regulating innate immune cell memory (see also Box 1).

**Box 1:In need of answers**

- How do innate immune cells sense metabolic states?
- What are the consequences of different metabolic environments on innate immune cell function and survival? Is innate immune metabolic reprogramming context dependent?
- Can our understanding of metabolic reprogramming in innate immune cells offer a potential therapeutic opportunity for the treatment of inflammatory diseases?

## Concluding remarks

Hypoxia is a prominent feature of inflammation associated with numerous disease states. Immune cells at hypoxic sites are required to maintain functional diversity and plasticity, as initially they have to adopt a pro‐inflammatory phenotype and then later switch to a pro‐resolution and repair phase. The regulation of immune metabolism by the HIF/hydroxylase pathway provides one mechanism by which innate immune cells can adapt to the hypoxic tissue environment. The immunological niche itself is likely to impact further on these metabolic adaptations, with evidence that small metabolites can in addition to their classical function act as signalling molecules regulating multiple cellular functions. This implicates impaired metabolism with impaired innate cell function. A better understanding of the metabolic states and sensing at the different inflammatory phases and the crosstalk with the HIF signalling pathway will be key to developing future therapeutic drugs for the treatment of diseases involving immune cells.

## Conflict of interest

The authors declare that they have no conflict of interest.

## References

[1] Walmsley SR , Print C , Farahi N , Peyssonnaux C , Johnson RS , Cramer T , Sobolewski A , Condliffe AM , Cowburn AS , Johnson N *et al* (2005) Hypoxia‐induced neutrophil survival is mediated by HIF‐1alpha‐dependent NF‐kappaB activity. J Exp Med 201: 105–115 15630139 10.1084/jem.20040624 PMC2212759

[2] Roiniotis J , Dinh H , Masendycz P , Turner A , Elsegood CL , Scholz GM , Hamilton JA (2009) Hypoxia prolongs monocyte/macrophage survival and enhanced glycolysis is associated with their maturation under aerobic conditions. J Immunol 182: 7974–7981 19494322 10.4049/jimmunol.0804216

[3] Hannah S , Mecklenburgh K , Rahman I , Bellingan GJ , Greening A , Haslett C , Chilvers ER (1995) Hypoxia prolongs neutrophil survival *in vitro* . FEBS Lett 372: 233–237 7556675 10.1016/0014-5793(95)00986-j

[4] Nissim Ben Efraim AH , Eliashar R , Levi‐Schaffer F (2010) Hypoxia modulates human eosinophil function. Clin Mol Allergy 8: 10 20642833 10.1186/1476-7961-8-10 PMC2923626

[5] Harris AJ , Thompson AR , Whyte MK , Walmsley SR (2014) HIF‐mediated innate immune responses: cell signaling and therapeutic implications. Hypoxia (Auckl) 2: 47–58 27774466 10.2147/HP.S50269 PMC5045056

[6] Chandel NS , Maltepe E , Goldwasser E , Mathieu CE , Simon MC , Schumacker PT (1998) Mitochondrial reactive oxygen species trigger hypoxia‐induced transcription. Proc Natl Acad Sci USA 95: 11715–11720 9751731 10.1073/pnas.95.20.11715 PMC21706

[7] Semenza GL , Jiang BH , Leung SW , Passantino R , Concordet JP , Maire P , Giallongo A (1996) Hypoxia response elements in the aldolase A, enolase 1, and lactate dehydrogenase A gene promoters contain essential binding sites for hypoxia‐inducible factor 1. J Biol Chem 271: 32529–32537 8955077 10.1074/jbc.271.51.32529

[8] Wang GL , Semenza GL (1996) Oxygen sensing and response to hypoxia by mammalian cells. Redox Rep 2: 89–96 27405946 10.1080/13510002.1996.11747034

[9] Maxwell PH , Pugh CW , Ratcliffe PJ (1993) Inducible operation of the erythropoietin 3’ enhancer in multiple cell lines: evidence for a widespread oxygen‐sensing mechanism. Proc Natl Acad Sci USA 90: 2423–2427 8460154 10.1073/pnas.90.6.2423 PMC46099

[10] Wang GL , Jiang BH , Rue EA , Semenza GL (1995) Hypoxia‐inducible factor 1 is a basic‐helix‐loop‐helix‐PAS heterodimer regulated by cellular O2 tension. Proc Natl Acad Sci USA 92: 5510–5514 7539918 10.1073/pnas.92.12.5510 PMC41725

[11] Kaelin WG Jr , Ratcliffe PJ (2008) Oxygen sensing by metazoans: the central role of the HIF hydroxylase pathway. Mol Cell 30: 393–402 18498744 10.1016/j.molcel.2008.04.009

[12] Epstein AC , Gleadle JM , McNeill LA , Hewitson KS , O'Rourke J , Mole DR , Mukherji M , Metzen E , Wilson MI , Dhanda A *et al* (2001) C. elegans EGL‐9 and mammalian homologs define a family of dioxygenases that regulate HIF by prolyl hydroxylation. Cell 107: 43–54 11595184 10.1016/s0092-8674(01)00507-4

[13] Lando D , Peet DJ , Whelan DA , Gorman JJ , Whitelaw ML (2002) Asparagine hydroxylation of the HIF transactivation domain a hypoxic switch. Science 295: 858–861 11823643 10.1126/science.1068592

[14] Coleman ML , Ratcliffe PJ (2009) Signalling cross talk of the HIF system: involvement of the FIH protein. Curr Pharm Des 15: 3904–3907 19671041 10.2174/138161209789649448

[15] Maxwell PH , Wiesener MS , Chang GW , Clifford SC , Vaux EC , Cockman ME , Wykoff CC , Pugh CW , Maher ER , Ratcliffe PJ (1999) The tumour suppressor protein VHL targets hypoxia‐inducible factors for oxygen‐dependent proteolysis. Nature 399: 271–275 10353251 10.1038/20459

[16] Jaakkola P , Mole DR , Tian YM , Wilson MI , Gielbert J , Gaskell SJ , von Kriegsheim A , Hebestreit HF , Mukherji M , Schofield CJ *et al* (2001) Targeting of HIF‐alpha to the von Hippel‐Lindau ubiquitylation complex by O2‐regulated prolyl hydroxylation. Science 292: 468–472 11292861 10.1126/science.1059796

[17] Schofield CJ , Ratcliffe PJ (2005) Signalling hypoxia by HIF hydroxylases. Biochem Biophys Res Commun 338: 617–626 16139242 10.1016/j.bbrc.2005.08.111

[18] Selak MA , Armour SM , MacKenzie ED , Boulahbel H , Watson DG , Mansfield KD , Pan Y , Simon MC , Thompson CB , Gottlieb E (2005) Succinate links TCA cycle dysfunction to oncogenesis by inhibiting HIF‐alpha prolyl hydroxylase. Cancer Cell 7: 77–85 15652751 10.1016/j.ccr.2004.11.022

[19] Hewitson KS , Lienard BM , McDonough MA , Clifton IJ , Butler D , Soares AS , Oldham NJ , McNeill LA , Schofield CJ (2007) Structural and mechanistic studies on the inhibition of the hypoxia‐inducible transcription factor hydroxylases by tricarboxylic acid cycle intermediates. J Biol Chem 282: 3293–3301 17135241 10.1074/jbc.M608337200

[20] Guitart AV , Panagopoulou TI , Villacreces A , Vukovic M , Sepulveda C , Allen L , Carter RN , van de Lagemaat LN , Morgan M , Giles P *et al* (2017) Fumarate hydratase is a critical metabolic regulator of hematopoietic stem cell functions. J Exp Med 214: 719–735 28202494 10.1084/jem.20161087 PMC5339674

[21] Tyrakis PA , Palazon A , Macias D , Lee KL , Phan AT , Velica P , You J , Chia GS , Sim J , Doedens A *et al* (2016) S‐2‐hydroxyglutarate regulates CD8(+) T‐lymphocyte fate. Nature 540: 236–241 27798602 10.1038/nature20165 PMC5149074

[22] Palsson‐McDermott EM , Curtis AM , Goel G , Lauterbach MA , Sheedy FJ , Gleeson LE , van den Bosch MW , Quinn SR , Domingo‐Fernandez R , Johnston DG *et al* (2015) Pyruvate kinase M2 regulates Hif‐1alpha activity and IL‐1beta induction and is a critical determinant of the warburg effect in LPS‐activated macrophages. Cell Metab 21: 65–80 25565206 10.1016/j.cmet.2014.12.005 PMC5198835

[23] Krzywinska E , Stockmann C (2018) Hypoxia, metabolism and immune cell function. Biomedicines 6: E56 29762526 10.3390/biomedicines6020056 PMC6027519

[24] Seagroves TN , Ryan HE , Lu H , Wouters BG , Knapp M , Thibault P , Laderoute K , Johnson RS (2001) Transcription factor HIF‐1 is a necessary mediator of the pasteur effect in mammalian cells. Mol Cell Biol 21: 3436–3444 11313469 10.1128/MCB.21.10.3436-3444.2001 PMC100265

[25] Papandreou I , Cairns RA , Fontana L , Lim AL , Denko NC (2006) HIF‐1 mediates adaptation to hypoxia by actively downregulating mitochondrial oxygen consumption. Cell Metab 3: 187–197 16517406 10.1016/j.cmet.2006.01.012

[26] Kim JW , Tchernyshyov I , Semenza GL , Dang CV (2006) HIF‐1‐mediated expression of pyruvate dehydrogenase kinase: a metabolic switch required for cellular adaptation to hypoxia. Cell Metab 3: 177–185 16517405 10.1016/j.cmet.2006.02.002

[27] Nizet V , Johnson RS (2009) Interdependence of hypoxic and innate immune responses. Nat Rev Immunol 9: 609–617 19704417 10.1038/nri2607 PMC4343208

[28] Cash TP , Pan Y , Simon MC (2007) Reactive oxygen species and cellular oxygen sensing. Free Radic Biol Med 43: 1219–1225 17893032 10.1016/j.freeradbiomed.2007.07.001 PMC2696222

[29] Knowles HJ , Mole DR , Ratcliffe PJ , Harris AL (2006) Normoxic stabilization of hypoxia‐inducible factor‐1alpha by modulation of the labile iron pool in differentiating U937 macrophages: effect of natural resistance‐associated macrophage protein 1. Cancer Res 66: 2600–2607 16510578 10.1158/0008-5472.CAN-05-2351

[30] Blouin CC , Page EL , Soucy GM , Richard DE (2004) Hypoxic gene activation by lipopolysaccharide in macrophages: implication of hypoxia‐inducible factor 1alpha. Blood 103: 1124–1130 14525767 10.1182/blood-2003-07-2427

[31] Cramer T , Yamanishi Y , Clausen BE , Forster I , Pawlinski R , Mackman N , Haase VH , Jaenisch R , Corr M , Nizet V *et al* (2003) HIF‐1alpha is essential for myeloid cell‐mediated inflammation. Cell 112: 645–657 12628185 10.1016/s0092-8674(03)00154-5 PMC4480774

[32] Peyssonnaux C , Datta V , Cramer T , Doedens A , Theodorakis EA , Gallo RL , Hurtado‐Ziola N , Nizet V , Johnson RS (2005) HIF‐1alpha expression regulates the bactericidal capacity of phagocytes. J Clin Invest 115: 1806–1815 16007254 10.1172/JCI23865 PMC1159132

[33] Thompson AA , Elks PM , Marriott HM , Eamsamarng S , Higgins KR , Lewis A , Williams L , Parmar S , Shaw G , McGrath EE *et al* (2014) Hypoxia‐inducible factor 2alpha regulates key neutrophil functions in humans, mice, and zebrafish. Blood 123: 366–376 24196071 10.1182/blood-2013-05-500207 PMC3894493

[34] Maianski NA , Geissler J , Srinivasula SM , Alnemri ES , Roos D , Kuijpers TW (2004) Functional characterization of mitochondria in neutrophils: a role restricted to apoptosis. Cell Death Differ 11: 143–153 14576767 10.1038/sj.cdd.4401320

[35] Fossati G , Moulding DA , Spiller DG , Moots RJ , White MR , Edwards SW (2003) The mitochondrial network of human neutrophils: role in chemotaxis, phagocytosis, respiratory burst activation, and commitment to apoptosis. J Immunol 170: 1964–1972 12574365 10.4049/jimmunol.170.4.1964

[36] Kempner W (1939) The nature of leukemic blood cells as determined by their metabolism. J Clin Invest 18: 291–300 16694664 10.1172/JCI101045 PMC434877

[37] McKinney GR , Martin SP , Rundles RW , Green R (1953) Respiratory and glycolytic activities of human leukocytes *in vitro* . J Appl Physiol 5: 335–340 13022600 10.1152/jappl.1953.5.7.335

[38] Tan AS , Ahmed N , Berridge MV (1998) Acute regulation of glucose transport after activation of human peripheral blood neutrophils by phorbol myristate acetate, fMLP, and granulocyte‐macrophage colony‐stimulating factor. Blood 91: 649–655 9427721

[39] Borregaard N , Herlin T (1982) Energy metabolism of human neutrophils during phagocytosis. J Clin Invest 70: 550–557 7107894 10.1172/JCI110647 PMC370256

[40] Boxer LA , Baehner RL , Davis J (1977) The effect of 2‐deoxyglucose on guinea pig polymorphonuclear leukocyte phagocytosis. J Cell Physiol 91: 89–102 856841 10.1002/jcp.1040910110

[41] Riffelmacher T , Clarke A , Richter FC , Stranks A , Pandey S , Danielli S , Hublitz P , Yu Z , Johnson E , Schwerd T *et al* (2017) Autophagy‐dependent generation of free fatty acids is critical for normal neutrophil differentiation. Immunity 47: 466–480.e5 28916263 10.1016/j.immuni.2017.08.005 PMC5610174

[42] Pithon‐Curi TC , Schumacher RI , Freitas JJ , Lagranha C , Newsholme P , Palanch AC , Doi SQ , Curi R (2003) Glutamine delays spontaneous apoptosis in neutrophils. Am J Physiol Cell Physiol 284: C1355–C1361 12529242 10.1152/ajpcell.00224.2002

[43] Stanton RC (2012) Glucose‐6‐phosphate dehydrogenase, NADPH, and cell survival. IUBMB Life 64: 362–369 22431005 10.1002/iub.1017 PMC3325335

[44] Cooper MR , DeChatelet LR , McCall CE , LaVia MF , Spurr CL , Baehner RL (1972) Complete deficiency of leukocyte glucose‐6‐phosphate dehydrogenase with defective bactericidal activity. J Clin Invest 51: 769–778 4401271 10.1172/JCI106871 PMC302189

[45] Bender JG , Van Epps DE (1985) Inhibition of human neutrophil function by 6‐aminonicotinamide: the role of the hexose monophosphate shunt in cell activation. Immunopharmacology 10: 191–199 3009354 10.1016/0162-3109(85)90025-6

[46] Brinkmann V , Reichard U , Goosmann C , Fauler B , Uhlemann Y , Weiss DS , Weinrauch Y , Zychlinsky A (2004) Neutrophil extracellular traps kill bacteria. Science 303: 1532–1535 15001782 10.1126/science.1092385

[47] Fuchs TA , Abed U , Goosmann C , Hurwitz R , Schulze I , Wahn V , Weinrauch Y , Brinkmann V , Zychlinsky A (2007) Novel cell death program leads to neutrophil extracellular traps. J Cell Biol 176: 231–241 17210947 10.1083/jcb.200606027 PMC2063942

[48] Kaplan MJ , Radic M (2012) Neutrophil extracellular traps: double‐edged swords of innate immunity. J Immunol 189: 2689–2695 22956760 10.4049/jimmunol.1201719 PMC3439169

[49] Rodriguez‐Espinosa O , Rojas‐Espinosa O , Moreno‐Altamirano MM , Lopez‐Villegas EO , Sanchez‐Garcia FJ (2015) Metabolic requirements for neutrophil extracellular traps formation. Immunology 145: 213–224 25545227 10.1111/imm.12437 PMC4427386

[50] Azevedo EP , Rochael NC , Guimaraes‐Costa AB , de Souza‐Vieira TS , Ganilho J , Saraiva EM , Palhano FL , Foguel D (2015) A metabolic shift toward pentose phosphate pathway is necessary for amyloid fibril‐ and phorbol 12‐myristate 13‐acetate‐induced neutrophil extracellular trap (NET) formation. J Biol Chem 290: 22174–22183 26198639 10.1074/jbc.M115.640094 PMC4571968

[51] Amini P , Stojkov D , Felser A , Jackson CB , Courage C , Schaller A , Gelman L , Soriano ME , Nuoffer JM , Scorrano L *et al* (2018) Neutrophil extracellular trap formation requires OPA1‐dependent glycolytic ATP production. Nat Commun 9: 2958 30054480 10.1038/s41467-018-05387-y PMC6063938

[52] Valentine WN , Follette JH , Lawrence JS (1953) The glycogen content of human leukocytes in health and in various disease states. J Clin Invest 32: 251–257 13044834 10.1172/JCI102734 PMC438336

[53] Scott RB (1968) Glycogen in human peripheral blood leukocytes. I. Characteristics of the synthesis and turnover of glycogen *in vitro* . J Clin Invest 47: 344–352 5638123 10.1172/JCI105730 PMC297176

[54] Robinson JM , Karnovsky ML , Karnovsky MJ (1982) Glycogen accumulation in polymorphonuclear leukocytes, and other intracellular alterations that occur during inflammation. J Cell Biol 95: 933–942 7153252 10.1083/jcb.95.3.933 PMC2112917

[55] Pescador N , Villar D , Cifuentes D , Garcia‐Rocha M , Ortiz‐Barahona A , Vazquez S , Ordonez A , Cuevas Y , Saez‐Morales D , Garcia‐Bermejo ML *et al* (2010) Hypoxia promotes glycogen accumulation through hypoxia inducible factor (HIF)‐mediated induction of glycogen synthase 1. PLoS One 5: e9644 20300197 10.1371/journal.pone.0009644 PMC2837373

[56] Sadiku P , Willson JA , Dickinson RS , Murphy F , Harris AJ , Lewis A , Sammut D , Mirchandani AS , Ryan E , Watts ER *et al* (2017) Prolyl hydroxylase 2 inactivation enhances glycogen storage and promotes excessive neutrophilic responses. J Clin Invest 127: 3407–3420 28805660 10.1172/JCI90848 PMC5669581

[57] Chan MC , Holt‐Martyn JP , Schofield CJ , Ratcliffe PJ (2016) Pharmacological targeting of the HIF hydroxylases–A new field in medicine development. Mol Aspects Med 47–48: 54–75 10.1016/j.mam.2016.01.001 26791432

[58] Jun HS , Weinstein DA , Lee YM , Mansfield BC , Chou JY (2014) Molecular mechanisms of neutrophil dysfunction in glycogen storage disease type Ib. Blood 123: 2843–2853 24565827 10.1182/blood-2013-05-502435 PMC4007611

[59] Kuijpers TW , Maianski NA , Tool AT , Smit GP , Rake JP , Roos D , Visser G (2003) Apoptotic neutrophils in the circulation of patients with glycogen storage disease type 1b (GSD1b). Blood 101: 5021–5024 12576310 10.1182/blood-2002-10-3128

[60] Kim SY , Jun HS , Mead PA , Mansfield BC , Chou JY (2008) Neutrophil stress and apoptosis underlie myeloid dysfunction in glycogen storage disease type Ib. Blood 111: 5704–5711 18420828 10.1182/blood-2007-12-129114 PMC2424162

[61] Madara JL , Patapoff TW , Gillece‐Castro B , Colgan SP , Parkos CA , Delp C , Mrsny RJ (1993) 5’‐adenosine monophosphate is the neutrophil‐derived paracrine factor that elicits chloride secretion from T84 intestinal epithelial cell monolayers. J Clin Invest 91: 2320–2325 8486793 10.1172/JCI116462 PMC288238

[62] Eltzschig HK , Eckle T , Mager A , Kuper N , Karcher C , Weissmuller T , Boengler K , Schulz R , Robson SC , Colgan SP (2006) ATP release from activated neutrophils occurs via connexin 43 and modulates adenosine‐dependent endothelial cell function. Circ Res 99: 1100–1108 17038639 10.1161/01.RES.0000250174.31269.70

[63] Harada Y , Kato Y , Miyaji T , Omote H , Moriyama Y , Hiasa M (2018) Vesicular nucleotide transporter mediates ATP release and migration in neutrophils. J Biol Chem 293: 3770–3779 29363573 10.1074/jbc.M117.810168 PMC5846168

[64] Chen Y , Corriden R , Inoue Y , Yip L , Hashiguchi N , Zinkernagel A , Nizet V , Insel PA , Junger WG (2006) ATP release guides neutrophil chemotaxis via P2Y2 and A3 receptors. Science 314: 1792–1795 17170310 10.1126/science.1132559

[65] Eltzschig HK , Ibla JC , Furuta GT , Leonard MO , Jacobson KA , Enjyoji K , Robson SC , Colgan SP (2003) Coordinated adenine nucleotide phosphohydrolysis and nucleoside signaling in posthypoxic endothelium: role of ectonucleotidases and adenosine A2B receptors. J Exp Med 198: 783–796 12939345 10.1084/jem.20030891 PMC2194189

[66] Eltzschig HK , Thompson LF , Karhausen J , Cotta RJ , Ibla JC , Robson SC , Colgan SP (2004) Endogenous adenosine produced during hypoxia attenuates neutrophil accumulation: coordination by extracellular nucleotide metabolism. Blood 104: 3986–3992 15319286 10.1182/blood-2004-06-2066

[67] Curtis VF , Cartwright IM , Lee JS , Wang RX , Kao DJ , Lanis JM , Burney KM , Welch N , Hall CHT , Goldberg MS *et al* (2018) Neutrophils as sources of dinucleotide polyphosphates and metabolism by epithelial ENPP1 to influence barrier function via adenosine signaling. Mol Biol Cell 29: 2687–2699 30188771 10.1091/mbc.E18-06-0377 PMC6249842

[68] Sica A , Mantovani A (2012) Macrophage plasticity and polarization: *in vivo* veritas. J Clin Invest 122: 787–795 22378047 10.1172/JCI59643 PMC3287223

[69] Jung S (2018) Macrophages and monocytes in 2017: macrophages and monocytes: of tortoises and hares. Nat Rev Immunol 18: 85–86 29292392 10.1038/nri.2017.158

[70] Wang T , Liu H , Lian G , Zhang SY , Wang X , Jiang C (2017) HIF1alpha‐induced glycolysis metabolism is essential to the activation of inflammatory macrophages. Mediators Inflamm 2017: 9029327 29386753 10.1155/2017/9029327 PMC5745720

[71] Fang HY , Hughes R , Murdoch C , Coffelt SB , Biswas SK , Harris AL , Johnson RS , Imityaz HZ , Simon MC , Fredlund E *et al* (2009) Hypoxia‐inducible factors 1 and 2 are important transcriptional effectors in primary macrophages experiencing hypoxia. Blood 114: 844–859 19454749 10.1182/blood-2008-12-195941 PMC2882173

[72] Imtiyaz HZ , Williams EP , Hickey MM , Patel SA , Durham AC , Yuan LJ , Hammond R , Gimotty PA , Keith B , Simon MC (2010) Hypoxia‐inducible factor 2alpha regulates macrophage function in mouse models of acute and tumor inflammation. J Clin Invest 120: 2699–2714 20644254 10.1172/JCI39506 PMC2912179

[73] Hard GC (1970) Some biochemical aspects of the immune macrophage. Br J Exp Pathol 51: 97–105 5434449 PMC2072214

[74] Newsholme P , Gordon S , Newsholme EA (1987) Rates of utilization and fates of glucose, glutamine, pyruvate, fatty acids and ketone bodies by mouse macrophages. Biochem J 242: 631–636 3593269 10.1042/bj2420631 PMC1147758

[75] Newsholme P , Curi R , Gordon S , Newsholme EA (1986) Metabolism of glucose, glutamine, long‐chain fatty acids and ketone bodies by murine macrophages. Biochem J 239: 121–125 3800971 10.1042/bj2390121 PMC1147248

[76] Xue J , Schmidt SV , Sander J , Draffehn A , Krebs W , Quester I , De Nardo D , Gohel TD , Emde M , Schmidleithner L *et al* (2014) Transcriptome‐based network analysis reveals a spectrum model of human macrophage activation. Immunity 40: 274–288 24530056 10.1016/j.immuni.2014.01.006 PMC3991396

[77] Biswas SK , Mantovani A (2010) Macrophage plasticity and interaction with lymphocyte subsets: cancer as a paradigm. Nat Immunol 11: 889–896 20856220 10.1038/ni.1937

[78] Takeda N , O'Dea EL , Doedens A , Kim JW , Weidemann A , Stockmann C , Asagiri M , Simon MC , Hoffmann A , Johnson RS (2010) Differential activation and antagonistic function of HIF‐{alpha} isoforms in macrophages are essential for NO homeostasis. Genes Dev 24: 491–501 20194441 10.1101/gad.1881410 PMC2827844

[79] Guentsch A , Beneke A , Swain L , Farhat K , Nagarajan S , Wielockx B , Raithatha K , Dudek J , Rehling P , Zieseniss A *et al* (2017) PHD2 is a regulator for glycolytic reprogramming in macrophages. Mol Cell Biol 37: e00236‐16 10.1128/MCB.00236-16 PMC5192080 27795296

[80] Takeda Y , Costa S , Delamarre E , Roncal C , Leite de Oliveira R , Squadrito ML , Finisguerra V , Deschoemaeker S , Bruyere F , Wenes M *et al* (2011) Macrophage skewing by Phd2 haplodeficiency prevents ischaemia by inducing arteriogenesis. Nature 479: 122–126 21983962 10.1038/nature10507 PMC4659699

[81] Lu L , Bonham CA , Chambers FG , Watkins SC , Hoffman RA , Simmons RL , Thomson AW (1996) Induction of nitric oxide synthase in mouse dendritic cells by IFN‐gamma, endotoxin, and interaction with allogeneic T cells: nitric oxide production is associated with dendritic cell apoptosis. J Immunol 157: 3577–3586 8871658

[82] Lorsbach RB , Murphy WJ , Lowenstein CJ , Snyder SH , Russell SW (1993) Expression of the nitric oxide synthase gene in mouse macrophages activated for tumor cell killing. Molecular basis for the synergy between interferon‐gamma and lipopolysaccharide. J Biol Chem 268: 1908–1913 7678412

[83] Vats D , Mukundan L , Odegaard JI , Zhang L , Smith KL , Morel CR , Wagner RA , Greaves DR , Murray PJ , Chawla A (2006) Oxidative metabolism and PGC‐1beta attenuate macrophage‐mediated inflammation. Cell Metab 4: 13–24 16814729 10.1016/j.cmet.2006.05.011 PMC1904486

[84] Doyle AG , Herbein G , Montaner LJ , Minty AJ , Caput D , Ferrara P , Gordon S (1994) Interleukin‐13 alters the activation state of murine macrophages *in vitro*: comparison with interleukin‐4 and interferon‐gamma. Eur J Immunol 24: 1441–1445 7911424 10.1002/eji.1830240630

[85] Mills CD , Kincaid K , Alt JM , Heilman MJ , Hill AM (2017) Pillars Article: M‐1/M‐2 Macrophages and the Th1/Th2 Paradigm. J. Immunol. 2000. 164: 6166–6173. J Immunol 199: 2194–2201 28923981 10.4049/jimmunol.1701141

[86] Warburg O , Wind F , Negelein E (1927) The metabolism of tumors in the body. J Gen Physiol 8: 519–530 19872213 10.1085/jgp.8.6.519 PMC2140820

[87] Tannahill GM , Curtis AM , Adamik J , Palsson‐McDermott EM , McGettrick AF , Goel G , Frezza C , Bernard NJ , Kelly B , Foley NH *et al* (2013) Succinate is an inflammatory signal that induces IL‐1beta through HIF‐1alpha. Nature 496: 238–242 23535595 10.1038/nature11986 PMC4031686

[88] Jha AK , Huang SC , Sergushichev A , Lampropoulou V , Ivanova Y , Loginicheva E , Chmielewski K , Stewart KM , Ashall J , Everts B *et al* (2015) Network integration of parallel metabolic and transcriptional data reveals metabolic modules that regulate macrophage polarization. Immunity 42: 419–430 25786174 10.1016/j.immuni.2015.02.005

[89] Koivunen P , Hirsila M , Remes AM , Hassinen IE , Kivirikko KI , Myllyharju J (2007) Inhibition of hypoxia‐inducible factor (HIF) hydroxylases by citric acid cycle intermediates: possible links between cell metabolism and stabilization of HIF. J Biol Chem 282: 4524–4532 17182618 10.1074/jbc.M610415200

[90] Mills EL , Kelly B , Logan A , Costa ASH , Varma M , Bryant CE , Tourlomousis P , Dabritz JHM , Gottlieb E , Latorre I *et al* (2016) Succinate dehydrogenase supports metabolic repurposing of mitochondria to drive inflammatory macrophages. Cell 167: 457–470.e13 27667687 10.1016/j.cell.2016.08.064 PMC5863951

[91] Haschemi A , Kosma P , Gille L , Evans CR , Burant CF , Starkl P , Knapp B , Haas R , Schmid JA , Jandl C *et al* (2012) The sedoheptulose kinase CARKL directs macrophage polarization through control of glucose metabolism. Cell Metab 15: 813–826 22682222 10.1016/j.cmet.2012.04.023 PMC3370649

[92] Nagy C , Haschemi A (2013) Sedoheptulose kinase regulates cellular carbohydrate metabolism by sedoheptulose 7‐phosphate supply. Biochem Soc Trans 41: 674–680 23514175 10.1042/BST20120354

[93] Aktan F (2004) iNOS‐mediated nitric oxide production and its regulation. Life Sci 75: 639–653 15172174 10.1016/j.lfs.2003.10.042

[94] Sbarra AJ , Karnovsky ML (1959) The biochemical basis of phagocytosis. I. Metabolic changes during the ingestion of particles by polymorphonuclear leukocytes. J Biol Chem 234: 1355–1362 13654378

[95] Rodriguez‐Prados JC , Traves PG , Cuenca J , Rico D , Aragones J , Martin‐Sanz P , Cascante M , Bosca L (2010) Substrate fate in activated macrophages: a comparison between innate, classic, and alternative activation. J Immunol 185: 605–614 20498354 10.4049/jimmunol.0901698

[96] Sag D , Carling D , Stout RD , Suttles J (2008) Adenosine 5’‐monophosphate‐activated protein kinase promotes macrophage polarization to an anti‐inflammatory functional phenotype. J Immunol 181: 8633–8641 19050283 10.4049/jimmunol.181.12.8633 PMC2756051

[97] Krawczyk CM , Holowka T , Sun J , Blagih J , Amiel E , DeBerardinis RJ , Cross JR , Jung E , Thompson CB , Jones RG *et al* (2010) Toll‐like receptor‐induced changes in glycolytic metabolism regulate dendritic cell activation. Blood 115: 4742–4749 20351312 10.1182/blood-2009-10-249540 PMC2890190

[98] Byles V , Covarrubias AJ , Ben‐Sahra I , Lamming DW , Sabatini DM , Manning BD , Horng T (2013) The TSC‐mTOR pathway regulates macrophage polarization. Nat Commun 4: 2834 24280772 10.1038/ncomms3834 PMC3876736

[99] Kimura T , Nada S , Takegahara N , Okuno T , Nojima S , Kang S , Ito D , Morimoto K , Hosokawa T , Hayama Y *et al* (2016) Polarization of M2 macrophages requires Lamtor1 that integrates cytokine and amino‐acid signals. Nat Commun 7: 13130 27731330 10.1038/ncomms13130 PMC5064021

[100] Degrossoli A , Arrais‐Silva WW , Colhone MC , Gadelha FR , Joazeiro PP , Giorgio S (2011) The influence of low oxygen on macrophage response to Leishmania infection. Scand J Immunol 74: 165–175 21517930 10.1111/j.1365-3083.2011.02566.x

[101] Cattin AL , Burden JJ , Van Emmenis L , Mackenzie FE , Hoving JJ , Garcia Calavia N , Guo Y , McLaughlin M , Rosenberg LH , Quereda V *et al* (2015) Macrophage‐induced blood vessels guide schwann cell‐mediated regeneration of peripheral nerves. Cell 162: 1127–1139 26279190 10.1016/j.cell.2015.07.021 PMC4553238

[102] Solinas G , Germano G , Mantovani A , Allavena P (2009) Tumor‐associated macrophages (TAM) as major players of the cancer‐related inflammation. J Leukoc Biol 86: 1065–1073 19741157 10.1189/jlb.0609385

[103] Murdoch C , Muthana M , Coffelt SB , Lewis CE (2008) The role of myeloid cells in the promotion of tumour angiogenesis. Nat Rev Cancer 8: 618–631 18633355 10.1038/nrc2444

[104] Netea MG , Joosten LA , Latz E , Mills KH , Natoli G , Stunnenberg HG , O'Neill LA , Xavier RJ (2016) Trained immunity: a program of innate immune memory in health and disease. Science 352: aaf1098 27102489 10.1126/science.aaf1098 PMC5087274

[105] Bekkering S , Arts RJW , Novakovic B , Kourtzelis I , van der Heijden C , Li Y , Popa CD , Ter Horst R , van Tuijl J , Netea‐Maier RT *et al* (2018) Metabolic induction of trained immunity through the mevalonate pathway. Cell 172: 135–146.e9 29328908 10.1016/j.cell.2017.11.025

[106] Cheng SC , Quintin J , Cramer RA , Shepardson KM , Saeed S , Kumar V , Giamarellos‐Bourboulis EJ , Martens JH , Rao NA , Aghajanirefah A *et al* (2014) mTOR‐ and HIF‐1alpha‐mediated aerobic glycolysis as metabolic basis for trained immunity. Science 345: 1250684 25258083 10.1126/science.1250684 PMC4226238

[107] Arts RJ , Novakovic B , Ter Horst R , Carvalho A , Bekkering S , Lachmandas E , Rodrigues F , Silvestre R , Cheng SC , Wang SY *et al* (2016) Glutaminolysis and fumarate accumulation integrate immunometabolic and epigenetic programs in trained immunity. Cell Metab 24: 807–819 27866838 10.1016/j.cmet.2016.10.008 PMC5742541

[108] Quintin J , Saeed S , Martens JHA , Giamarellos‐Bourboulis EJ , Ifrim DC , Logie C , Jacobs L , Jansen T , Kullberg BJ , Wijmenga C *et al* (2012) Candida albicans infection affords protection against reinfection via functional reprogramming of monocytes. Cell Host Microbe 12: 223–232 22901542 10.1016/j.chom.2012.06.006 PMC3864037

[109] Losman JA , Kaelin WG Jr (2013) What a difference a hydroxyl makes: mutant IDH, (R)‐2‐hydroxyglutarate, and cancer. Genes Dev 27: 836–852 23630074 10.1101/gad.217406.113 PMC3650222

[110] Burr SP , Costa AS , Grice GL , Timms RT , Lobb IT , Freisinger P , Dodd RB , Dougan G , Lehner PJ , Frezza C *et al* (2016) Mitochondrial protein lipoylation and the 2‐oxoglutarate dehydrogenase complex controls HIF1alpha stability in aerobic conditions. Cell Metab 24: 740–752 27923773 10.1016/j.cmet.2016.09.015 PMC5106373

[111] Thompson AA , Dickinson RS , Murphy F , Thomson JP , Marriott HM , Tavares A , Willson J , Williams L , Lewis A , Mirchandani A *et al* (2017) Hypoxia determines survival outcomes of bacterial infection through HIF‐1alpha dependent re‐programming of leukocyte metabolism. Sci Immunol 2: eaal2861 28386604 10.1126/sciimmunol.aal2861 PMC5380213

